# Introgression of *Aegilops speltoides* segments in *Triticum aestivum* and the effect of the gametocidal genes

**DOI:** 10.1093/aob/mcx149

**Published:** 2017-12-05

**Authors:** Julie King, Surbhi Grewal, Cai-yun Yang, Stella Hubbart Edwards, Duncan Scholefield, Stephen Ashling, John A Harper, Alexandra M Allen, Keith J Edwards, Amanda J Burridge, Ian P King

**Affiliations:** 1 Division of Plant and Cop Sciences, University of Nottingham, Sutton Bonington Campus, Loughborough, UK; 2 The Institute of Biological, Environmental and Rural Sciences, Aberystwyth University, Plas Gogerddan, UK; 3 School of Biological Sciences, University of Bristol, UK

**Keywords:** *Aegilops speltoides*, *Triticum aestivum*, introgression, recombination, comparative synteny, gametocidal, genetic linkage mapping, GISH, chromosome transmission

## Abstract

**Background and Aims:**

Bread wheat (*Triticum aestivum*) has been through a severe genetic bottleneck as a result of its evolution and domestication. It is therefore essential that new sources of genetic variation are generated and utilized. This study aimed to generate genome-wide introgressed segments from *Aegilops speltoides*. Introgressions generated from this research will be made available for phenotypic analysis.

**Methods:**

*Aegilops speltoides* was crossed as the male parent to *T. aestivum* ‘Paragon’. The interspecific hybrids were then backcrossed to Paragon. Introgressions were detected and characterized using the Affymetrix Axiom Array and genomic *in situ* hybridization (GISH).

**Key Results:**

Recombination in the gametes of the *F*_1_ hybrids was at a level where it was possible to generate a genetic linkage map of *Ae. speltoides*. This was used to identify 294 wheat/*Ae. speltoides* introgressions. Introgressions from all seven linkage groups of *Ae. speltoides* were found, including both large and small segments. Comparative analysis showed that overall macro-synteny is conserved between *Ae. speltoides* and *T. aestivum*, but that *Ae. speltoides* does not contain the 4A/5A/7B translocations present in wheat. *Aegilops speltoides* has been reported to carry gametocidal genes, i.e. genes that ensure their transmission through the gametes to the next generation. Transmission rates of the seven *Ae. speltoides* linkage groups introgressed into wheat varied. A 100 % transmission rate of linkage group 2 demonstrates the presence of the gametocidal genes on this chromosome.

**Conclusions:**

A high level of recombination occurs between the chromosomes of wheat and *Ae. speltoides*, leading to the generation of large numbers of introgressions with the potential for exploitation in breeding programmes. Due to the gametocidal genes, all germplasm developed will always contain a segment from *Ae. speltoides* linkage group 2S, in addition to an introgression from any other linkage group.

## INTRODUCTION

Bread wheat (*Triticum aestivum*), one of the world’s leading sources of food, is an allopolyploid (6*x* = AABBDD = 42) composed of the genomes of three different species. The A genome is derived from *Triticum urartu* (Dvorak *et al.*, 1993), the B from *Aegilops speltoides* or a closely related species (Sarkar and Stebbins, 1956; Riley *et al.*, 1958; Dvorak and Zhang, 1990; Maestra and Naranjo, 1998; Marcussen *et al.*, 2014) and the D genome from *Aegilops tauschii* (McFadden and Sears, 1946). The final hybridization event between tetraploid wheat (AABB) and *Ae. tauschii* (DD) is thought to have occurred only once or twice, ~8000–10 000 years ago. As a result, wheat went through a significant genetic bottleneck. Thus, the significant yield gains achieved by wheat breeders to date have been via the exploitation of genetic variation that has arisen via gene mutation over the last 8000–10 000 years and rare outcrossing events with tetraploid wheat. In many parts of the world wheat yields are now starting to plateau and this is thought to be a direct result of a lack of genetic variation compounded by the changing environment at a time of increasing demand for food due to the increasing global population (Charmet, 2011; Grassini *et al.*, 2013; Ray *et al.*, 2013). It is therefore essential that new sources of genetic variation be found that will enable breeders to generate the next generation of high-yielding environmentally adapted wheat varieties (Tanksley and McCouch, 1997; Haussmann *et al.*, 2004; Tester and Langridge, 2010; McCouch *et al.*, 2013; Warschefsky *et al.*, 2014; Brozynska *et al.*, 2015; Zhang *et al.*, 2017)

Unlike wheat, its progenitor species and wild relatives provide a vast and largely untapped source of genetic variation for most, if not all, traits of agronomic importance. Considerable efforts have been made to exploit the genetic variation within the progenitors and wild relatives, with some noticeable successes, for example the transfer of a segment of *Aegilops umbellulata* to wheat conferring resistance to leaf rust (Sears, 1955), the transfer of a segment from *Aegilops ventricosa* carrying resistance to eyespot (Maia, 1967; Doussinault *et al.*, 1983) and its subsequent release in the variety Rendevouz, and the registration of ‘MACE’, a hard red winter wheat carrying a segment from *Thinopyrum intermedium* conferring resistance to wheat streak mosaic virus (Graybosch *et al.*, 2009). Progress has been severely hindered, however, due to an inability to quickly and accurately identify and characterize interspecific transfers to wheat. However, with the advent of new molecular technologies coupled with specific crossing strategies, we can now systematically exploit the genetic variation available in the progenitors of wheat and its wild relatives. (e.g. Tiwari et al., 2014, 2015; King et al., 2016, 2017).

The transfer of genetic variation to wheat from its progenitors/wild relatives occurs via recombination between the chromosomes of wheat and those of its wild relative at meiosis in *F*_1_ hybrids or in their derivatives, e.g. addition and substitution lines (King et al., 2016, 2017). This results in the generation of interspecific recombinant chromosomes. Once identified, interspecific recombinant chromosomes are then recurrently backcrossed with wheat. The ultimate objective is to generate lines of wheat carrying a small chromosome segment from a progenitor/wild relative with a target gene but few, if any, deleterious genes.

One of the wild relatives of wheat that is of particular interest to researchers is *Ae. speltoides*, a rich source of genetic variation for resistance to a range of diseases of importance in wheat (Jia *et al.*, 1996; Mago *et al.*, 2009; Klindworth *et al.*, 2012; Deek and Distelfeld, 2014). *Aegilops speltoides*, or an extinct close relative, has been proposed as the donor of the B genome of wheat (Sarkar and Stebbins, 1956; Dvorak and Zhang, 1990; Friebe and Gill, 1996; Tsunewaki, 1996; Dvorak and Akhunov, 2005; Marcussen *et al.*, 2014). *Aegilops speltoides* is a facultative outbreeder, but can be readily inbred in the glasshouse.

Unlike most of the wild relatives of wheat, *Ae. speltoides* possesses the ability to suppress the wheat *Ph1* locus (Dvorak, 1972; Dvorak *et al.*, 2006). Recombination is normally restricted to homologous chromosomes from the same genome, i.e. although the A, B and D genomes of wheat and chromosomes from wild relatives are related, recombination can only occur within genomes in the presence of *Ph1*. However, if *Ph1* is removed, related chromosomes (homoeologues) from different genomes can recombine at meiosis. *Aegilops speltoides* possesses genes on chromosomes 3S (*Su1-Ph1*) and 7S (*Su2-Ph1*) (Dvorak *et al.*, 2006), with the ability to suppress *Ph1*. Thus, in *F*_1_ hybrids between wheat and *Ae. speltoides Su1-Ph1* or *Su2-Ph1* enables intergenomic homoeologous recombination to occur during meiosis.

*Aegilops speltoides* also carries gametocidal genes (*Gc*) (Tsujimoto and Tsunewaki, 1984, 1988; Ogihara *et al.*, 1994), which are preferentially transmitted to the next generation. Individuals heterozygous for *Gc* gene(s) produce two types of gametes. Both male and female gametes that lack the *Gc* genes are generally not viable due to chromosome breakage (Finch *et al.*, 1984). Gametes that carry the *Gc* genes, however, behave normally and thus it is only these gametes that are transmitted to the next generation. *Aegilops speltoides* carries two alleles of the gametocidal genes. *Gc1a* and *Gc1b*, both located on chromosome 2S, are transmitted at a very high frequency to the next generation and give rise to different morphological aberrations. *Gc1a* causes endosperm degeneration and chromosome aberrations while *Gc1b* results in the production of seeds that lack shoot primordia (Tsujimoto and Tsunewaki, 1988). The limitations of utilizing species with gametocidal genes for wild relative introgression are: (1) the fertility of hybrid/crossed grain is reduced by at least 50 %; and (2) all introgressions from wild relatives with *Gc* genes will always carry them and any other genes closely linked to them. Hence it is very difficult, if not impossible, to transfer genes from other regions of the genome in the absence of the *Gc* genes.

In this paper we investigate (1) the ability of the *Ae. speltoides* suppressors of the *Ph1* locus located on chromosomes 3S and 7S to induce recombination between the chromosomes of wheat and those of *Ae. speltoides*, and (2) the frequency of transmission of the *Ae. speltoides* gametocidal chromosomes through the gametes in *F*_1_ hybrids and their derivatives.

## MATERIALS AND METHODS

### Generation of introgressions

**Fig. 1.**
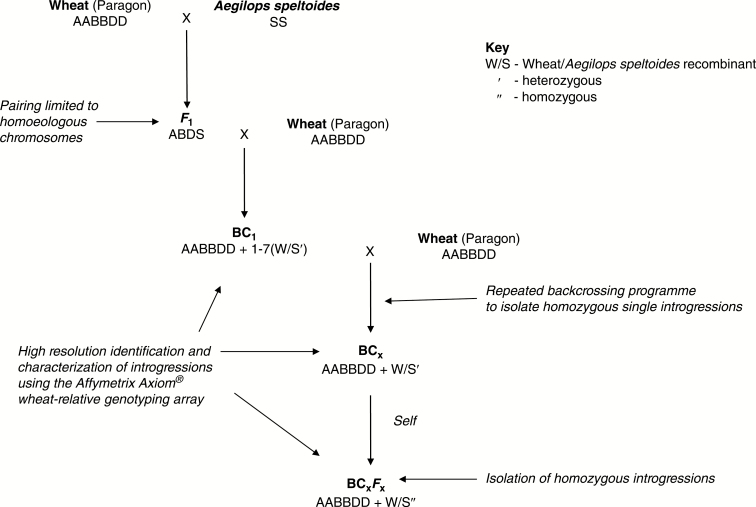
Wheat/wild relative introgression strategy.

The method used to induce introgressions between wheat and *Ae. speltoides* is the same as that described by King *et al.* (2017). In summary, hexaploid wheat (‘Paragon’, obtained from the Germplasm Resources Unit at the JIC; code W10074) was pollinated with *Ae. speltoides* (accession 2140066 obtained from the Germplasm Resources Unit at the JIC) to produce *F*_1_ interspecific hybrids. These hybrids were then grown to maturity and backcrossed as the female with the wheat parent to generate BC_1_ populations. The BC_1_ individuals and their resulting progenies were then recurrently pollinated to produce BC_2,_ BC_3_ etc. populations(Fig. 1).

As a result of the presence of the suppressors of the *Ph1* locus, homoeologous recombination was expected to occur at meiosis in the *F*_1_ wheat/*Ae. speltoides* hybrids, leading to the production of interspecific recombinant chromosomes/introgressions. Subsequent recurrent backcrossing of any BC_1_ progeny to the wheat parent was undertaken to transfer any interspecific recombinant chromosomes generated into a wheat (Paragon) background.

### Detection of putative wheat/*Ae. speltoides* introgressions

A 35K Axiom^®^ Wheat-Relative Genotyping Array (Affymetrix, Santa Clara, CA) was used to detect the presence of putative wheat/*Ae. speltoides* introgressions in each of the backcross generations (King *et al.*, 2017). In summary, the array is composed of single-nucleotide polymorphisms (SNPs), each showing polymorphism for the ten wild relatives (under study at the Nottingham/BBSRC Wheat Research Centre), including *Ae. speltoides* (King *et al.*, 2017). All the SNPs incorporated in this array formed part of the Axiom^®^ 820K SNP array (Winfield *et al.*, 2016). The dataset for the Axiom^®^ 820K SNP array is available from www.cerealsdb.uk.net (Wilkinson et al., 2012, 2016). Table 1 shows the number of putative SNPs between *Ae. speltoides* and each of the wheat genotypes included on the array. The array has been constructed in such a way that up to 384 lines could be screened at one time. Thus, the array facilitated the high-throughput, high-resolution screening of wheat/*Ae. speltoides* introgressions. Genotyping was performed as described by King *et al.* (2017) with no modifications (see below).

**Table 1.** Number of polymorphic SNPs between *Ae. speltoides* and hexaploid wheat based on the Affymetrix 35K array. A comparison is made between all calls (all markers showing polymorphism between wheat and *Ae. speltoides*) and Poly High Resolution (PHR) calls, which are codominant and polymorphic between wheat and *Ae. speltoides* with at least two examples of the minor allele

	Linkage group							Total
	1	2	3	4	5	6	7	
All calls (% of total)	2770 (12.4)	3992 (17.9)	3358 (15.1)	2798 (12.6)	3646 (16.4)	2530 (11.4)	3164 (14.2)	22 258
PHR calls (% of total)	69 (12.7)	78 (14.3)	82 (15.1)	85 (15.6)	90 (16.5)	46 (8.5)	94 (17.3)	544

### Genetic mapping of *Ae. speltoides*

**Fig. 2.**
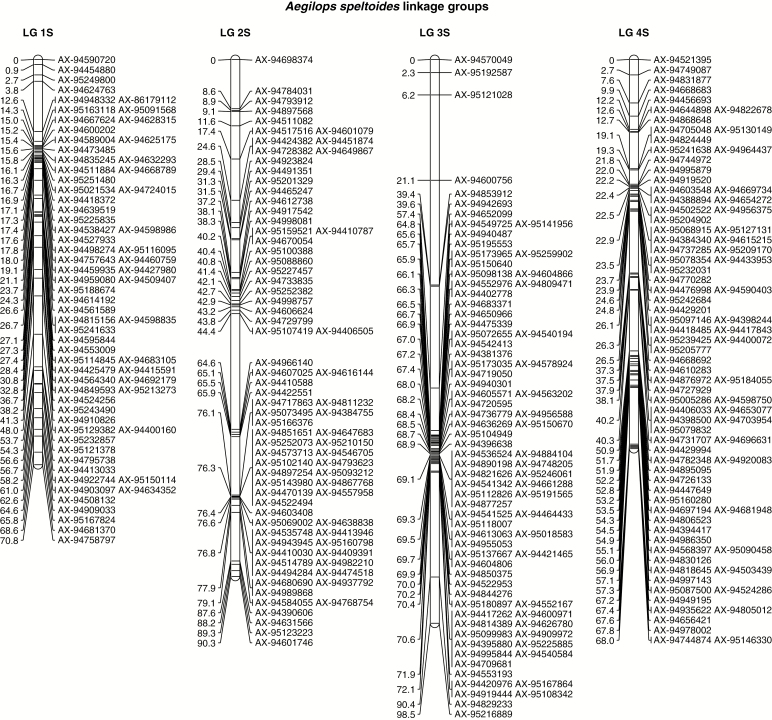

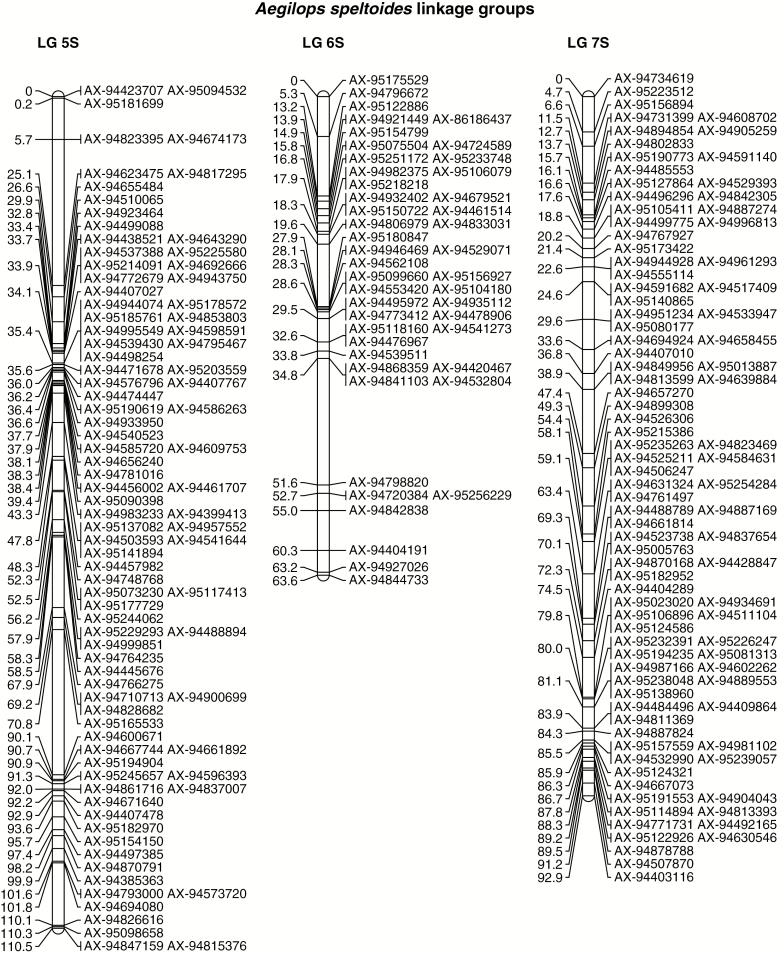
Genetic linkage map of *Ae. speltoides*.

Genetic mapping was as described by King *et al.* (2017); in summary, DNA was extracted using a CTAB method (Zhang *et al.*, 2013) from individuals of the back-crossed populations, BC_1_, BC_2_, BC_3,_ BC_4_ and BC_5_ derived from the wheat/*Ae. speltoides F*_1_ hybrids. These populations were genotyped with the Axiom^®^ Wheat-Relative Genotyping Array. Only Poly High Resolution (PHR) SNP markers, which were codominant and polymorphic, with at least two examples of the minor allele were used for genetic mapping (King *et al.*, 2017). The SNP markers that showed (1) heterozygous calls for either parent(s), (2) no polymorphism between the wheat parents and *Ae. speltoides*, and/or (3) no calls for either parent(s) were removed using Flapjack™ (Milne *et al.*, 2010; v.1.14.09.24). The resulting markers were sorted into linkage groups (Fig. 2) in JoinMap^®^ 4.1 (van Ooijen, 2011) with a LOD score of 20 and a recombination frequency threshold of 0.1 using the Haldane mapping function (Haldane, 1919). All markers that did not show any heterozygous call or were unlinked were ignored and only the highest-ranking linkage groups with >30 markers were selected for map construction. These were exported and assigned to chromosomes using information from the Axiom^®^ Wheat HD Genotyping Array (Winfield *et al.*, 2016).

### Comparative analysis

**Fig. 3.**
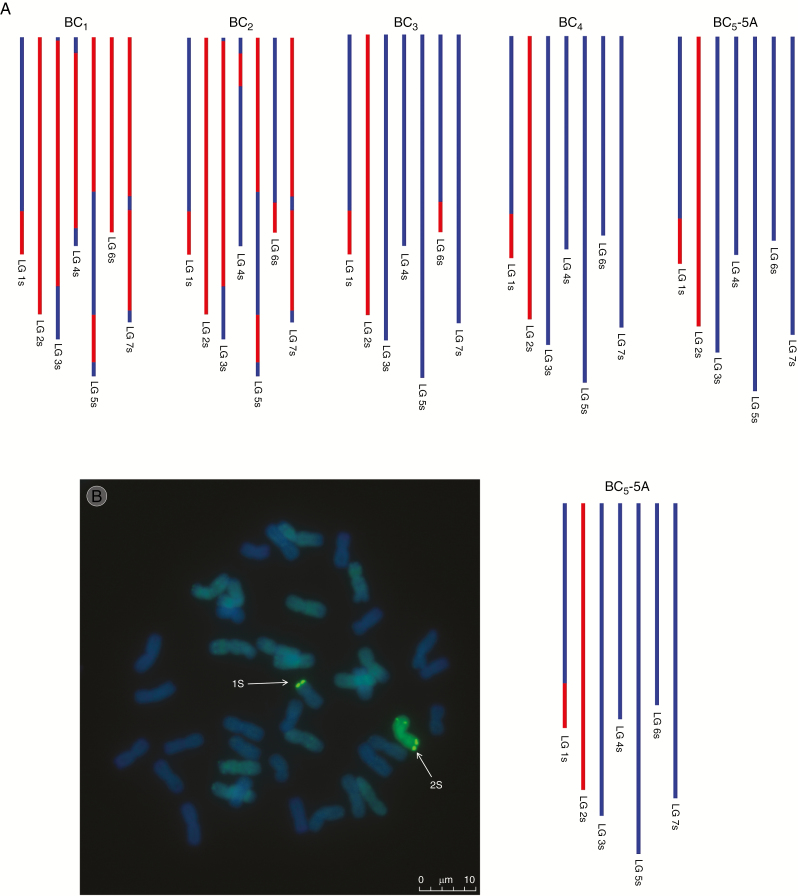
(A) SNP characterization of *Ae. speltoides* introgressions in five consecutive generations, i.e. BC_1_, BC_2_, BC_3_, BC_4_ and BC_5_. (B) Genomic *in situ* hybridization image of the BC_5_ genotype (aneuploid 41-chromosome plant). In the SNP characterization red colour is used to represent the presence of an *Ae. speltoides* introgression and blue colour represents wheat. It should be noted that these diagrams cannot be used to assess which wheat chromosomes the *Ae. speltoides* segments have recombined with. The GISH image shows a metaphase spread of BC_5_-5A probed with labelled genomic DNA of *Ae. speltoides*. Arrows show *Ae. speltoides* introgressions (green).

Synteny analysis was carried out using sequence information of the markers located on the present map of *Ae. speltoides*. The sequences of the mapped markers were compared using BLAST (e-value cut-off of 1e-05) against the wheat genome (http://plants.ensembl.org/Triticum_aestivum) to obtain the orthologous map positions of the top hits in the A, B and D genomes of wheat. To generate the figures, centimorgan (cM) distances on the linkage groups of the present map of *Ae. speltoides* were scaled up by a factor of 100 000 to match similar base-pair lengths of the chromosomes of the wheat genome. Figure 3 was visualized using Circos (v. 0.67; Krzywinski *et al.*, 2009) in order to observe synteny between *Ae. speltoides* (genetic position in cM) and the wheat genome (physical position in Mb).

### Cytogenetic analysis

The protocol for genomic *in situ* hybridization (GISH) was as described in Zhang *et al.* (2013), Kato *et al.* (2004) and King *et al.* (2017). In summary, genomic DNAs from young leaves of the three putative diploid progenitors of bread wheat, i.e. *T. urartu* (A genome), *Ae. speltoides* (B genome) and *Ae. tauschii* (D genome), were isolated using extraction buffer (0.1 m Tris–HCl pH 7.5, 0.05 m EDTA pH 8.0, 1.25% SDS). Samples were incubated at 65 °C for 1 h before being placed on ice and mixed with ice-cold 6 m NH_4_C_2_H_3_O for 15 min. The samples were then spun down, the supernatant was mixed with isopropanol to pellet the DNA and the isolated DNA was further purified with phenol/chloroform. The genomic DNAs of *Ae. speltoides* and *T. urartu* were labelled by nick translation with Chroma Tide Alexa Fluor 488-5-dUTP (Invitrogen, Carlsbad, CA; C11397). Genomic DNA of *Ae. tauschii* was labelled with Alexa Fluor 594-5-dUTP (Invitrogen; C11400). Genomic DNAs of *Ae. speltoides* and *T. aestivum* ‘Chinese Spring’ were fragmented to 300–500 bp in a heat block at 100 °C.

Preparation of chromosome spreads was as described in Kato *et al.* (2004) and King *et al.* (2017). Briefly, roots were excised from germinated seeds, treated with nitrous oxide gas at 10 bar for 2 h, fixed in 90 % acetic acid for 10 min and then washed three times in water on ice. Root tips were dissected and digested in 20 µL of 1 % pectolyase Y23 and 2 % cellulase Onozuka R-10 (Yakult Pharmaceutical, Tokyo) solution for 50 min at 37 °C and then washed three times in 70 % ethanol. Root tips were crushed in 70 % ethanol, cells collected by centrifugation at 2.5 *g* for 1 min, briefly dried and then re-suspended in 30–40 µL of 100 % acetic acid prior to being placed on ice. The cell suspension was dropped onto glass slides (6–7 µL per slide) in a moist box and dried slowly under cover.

Slides were initially probed using labelled genomic DNA of *Ae. speltoides* (100 ng) and fragmented genomic DNA of Chinese Spring (3000 ng) as blocker (in a ratio of 1: 30 per slide) to detect the *Ae. speltoides* introgressions. The slides were bleached (dipped in 2 × saline–sodium citrate (SSC) to remove the coverslip, transferred to 4 × SSC for 5 min and air-dried in the light) and re-probed with labelled DNAs of *T. urartu* (100 ng) and *Ae. tauschii* (200 ng) and fragmented DNA of *Ae. speltoides* (3000 ng) as blocker in a ratio of 1: 2:30 per slide to detect the AABBDD genomes of wheat. In both cases, the hybridization mix was made up to 10 µL with 2 × SSC in 1 × Tris/EDTA buffer (TE). Slides were incubated initially at 75 °C for 5 min and then overnight at 55 °C in a closed box before counterstaining with Vectashield mounting medium with 4',6-diamidino-2-phenylindole,dihydrochloride (DAPI), and analysed using a high-throughput, fully automated Zeiss Axio Imager Z2 upright epifluorescence microscope (Carl Zeiss, Oberkochen, Germany) with filters for DAPI (blue), Alexa Fluor 488 (green) and Alexa Fluor 594 (red). Photographs were taken using a MetaSystems Coolcube 1m CCD camera. Further slide analysis was carried out using Meta Systems ISIS and Metafer software (Metasystems, Altlussheim, Germany). This system enabled the fully automated capture of high- and low-power fluorescent images of root tip metaphase spreads. Slides with root tip preparations were automatically scanned and the images downloaded for analysis.

## RESULTS

### Generation of wheat/*Ae. speltoides* introgressions

A total of 3890 crosses were made between wheat and *Ae. speltoides* and their derivatives, leading to the generation of 9953 crossed seeds and 2029 self-seeds (Fig. 1). The number of seeds germinated, plants crossed and seed set are shown in Table 2. Every ear produced by the *F*_1_ was crossed with wheat. The *F*_1_ hybrids generated between wheat and *Ae. speltoides* showed the lowest frequency of germination. Only 15 % germinated compared with 100, 73, 70 and 78 % in the BC_1_, BC_2_, BC_3_ and BC_4_ generations. The *F*_1_ hybrids also showed the lowest fertility, with only 29 % of crossed ears producing any seeds compared with crossed ears from the BC_1_, BC_2_, BC_3_ and BC_5_ generations, which showed 58, 72, 83 and 84 % fertility, respectively.

**Table 2.** Number of seed produced and germinated in relation to the number of crosses carried out for each generation of the introgression programme for *Ae. speltoides* into wheat

	Wheat × *Ae. speltoides*	*F*_1_	BC_1_	BC_2_	BC_3_	BC_4_	Total
Number of seeds sown	NA	20	22	400	187	173	802
Number of seeds that germinated (%)	NA	3 (15)	22 (100)	292 (73)	130 (70)	135 (78)	582
Number of plants that set seed (%)	NA	3 (100)	22 (100)	261 (89)	124 (95)	117 (87)	527
Number of seed/total number of crosses (average number of seed set per crossed ear)	127/26 (4.9)	22/35 (0.6)	509/357 (1.4)	5743/2180 (2.6)	3204/876 (3.7)	348/416 (0.8)	9963/3890
Number of crosses producing seed (%)	18 (69)	10 (29)	207 (58)	1579 (72)	730 (83)	350 (84)	2894
Number of self seed produced	0	0	21	520	753	735	2029

Of the 20 *F*_1_ seeds germinated, only three reached maturity and set seeds. Thus all of the wheat/*Ae. speltoides* introgressions generated in this programme were limited to these three viable *F*_1_ hybrids. Table 2 summarizes the number of seeds germinated and seed set for each of the backcross generations. In each generation, the average seed set per crossed ear was very low, i.e. the lowest average seed set per ear was 0.6 (observed in the *F*_1_ hybrid), while the highest average seed set per crossed ear was only 3.7 (observed in the BC_3_). The fertility of plants that had previously been self-fertilized at least once was considerably higher than that of plants in the backcross programme, although again variability was seen, e.g. BC_3_*F*_1_-16A was male-sterile and therefore produced no self-fertilized seed, while seed numbers from crossed ears ranged from 8 to 19. Some plants were also found to be asynchronous for anther and stigma maturity. For these plants with manual pollination, self-fertilized seeds were produced.

### Detection of introgressions

Of the SNPs on the Axiom array, 22 258 were polymorphic between *Ae. speltoides* and wheat (Table 1). The Axiom array was used to screen genomic DNA prepared from 536 samples of BC_1_ to BC_5_ lines between wheat and *Ae. speltoides*. Genotype calls were generated, and the sample call rate (markers working in a particular genotype) ranged from 86.5 to 99.8 %, with an average of 98.8 % for the 536 samples. Affymetrix software classified the scores for each SNP into one of six cluster patterns. However, only those classified as Poly High Resolution (PHR) were used for genetic mapping as these are considered to be optimum quality. Linkage group 7 had the highest number of SNPs (17.3 %), while linkage group 6 had the lowest number (8.5 %).

JoinMap^®^ (van Ooijen, 2011) was used to analyse the PHR SNPs and this led to the establishment of seven linkage groups. The genetic map was composed of 544 SNPs and represented the seven chromosomes of *Ae. speltoides.* Linkage groups 1–7 had 69, 78, 82, 85, 90, 46 and 94 SNPs respectively (Fig. 2). The lengths of linkage groups 1–7 were 70.8, 90.3, 98.5, 68, 110.5, 63.6 and 92.9 cM, respectively, with a total length of 594.6 cM and an average chromosome length of 84.9 cM.

### Genomic *in situ* hybridization

In most cases the number of wheat/*Ae. speltoides* introgressions detected by genomic *in situ* hybridization (GISH) in BC_4_ and BC_5_ individuals corresponded exactly with the number detected via SNP analysis (Table 3 and Fig. 3). One exception was detected in the GISH analysis of BC_5_-4A. The SNP analysis of this plant showed the only segment present to be a complete 2S chromosome. However, the GISH image showed the presence of a large *Ae. speltoides* 2S segment (one telomere was missing from the chromosome) and a very small *Ae. speltoides* segment recombined with a different wheat chromosome.

**Table 3.** Number of introgressed segments from *Ae. speltoides* present in BC_4_ and BC_5_ plants as detected by SNP genotyping and GISH. The *Ae. speltoides* chromosomes have been assigned to linkage groups via the comparative analysis of the SNPs with wheat

Plant accession numbers		Number of segments		*Ae. speltoides* linkage group of segments
Backcross	Number	Genotyping	GISH	
BC_4_ BC_5_	45A	3	3	2, 5, 6
	45C	1	1	2
	45D	3	3	2, 5, 6
	1A	3	3	2, 4, 5
	1B	1	1	2
	1C	1	1	2
	1D	2	2	2, 5
	2	2	2	2, 5
	3	1	1	1
	4A	1	1 + small end	2,?
	5A	2	2	1, 2
	6A	1	1	2
	6B	2	2	1, 2
	6C	1	1	2
	7A	2	2	2, 5
	7C	1	1	2
	8A	1	1	2
	8B	1	1	2
	8C	1	1	2
	8D	1	1	2

### Syntenic relationship

**Fig. 4.**
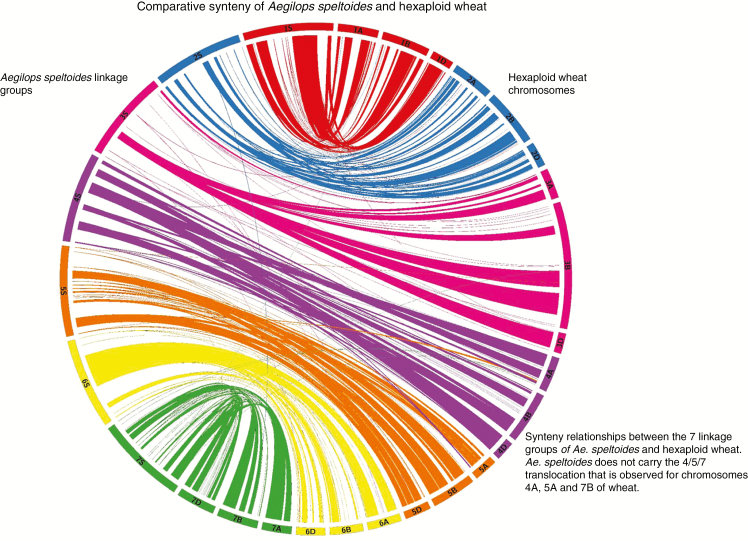
Synteny of *Ae. speltoides* (genetic position in cM) with hexaploid wheat (physical position in Mb) [visualized using Circos v. 0.67 (Krzywinski *et al.*, 2009)].

Figure 4 shows the syntenic relationship between the seven linkage groups of *Ae. speltoides* and the three genomes of wheat, with large ribbons showing significant synteny. Some gene rearrangements are indicated where single markers cross-map to non-collinear positions on wheat chromosomes. The only major disruption in synteny between the two species is that *Ae. speltoides* does not carry the 4/5/7 translocation observed for chromosomes 4A, 5A and 7B of wheat (Liu *et al.*, 1992; Naranjo *et al.*, 1987). These data demonstrate that there is a close syntenic relationship between the A, B and D genomes of wheat and *Ae. speltoides*.

### Preferential transmission

**Fig. 5.**
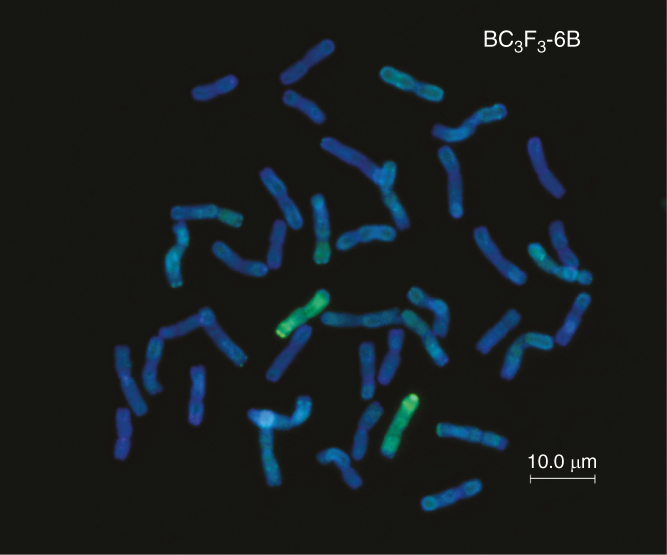
GISH of a complete one-cell metaphase spread of a genotype produced by self-fertilization (BC_3_F_3_-6B) with labelled genomic *Ae. speltoides* DNA as probe, showing a homozygous pair of *Ae. speltoides* linkage group 2 introgressed chromosomes (green).

The data obtained from SNP analysis was used to determine the transmission frequency of chromosome segments from each of the seven *Ae. speltoides* linkage groups through the female gametes to the BC_1_, BC_2_, BC_3_, BC_4_ and BC_5_ generations. Chromosome segments from linkage group 2 were observed in all 536 backcross progeny observed (Table 4). The smallest segment carried only three SNP markers, AX94631566, AX95123223 and AX94601746, located near one of the terminal ends of the 2S linkage group (Fig. 2). These three SNP markers were found to be present in all the backcross individuals analysed. In order to determine whether the 2S chromosome was also preferentially transmitted through the male gametes, 41 BC_3_ and BC_4_ plants were allowed to self-fertilize and their progenies were analysed using GISH. The BC_3_/BC_4_ plants selected each carried a single copy of a 2S chromosome segment. Thus, these plants would produce two types of male and female gametes during gametogenesis: those that carried 2S and those that did not. If the 2S chromosome segment was preferentially transmitted through both the male and female gametes, only those that carried the 2S chromosome would be viable and as a result all the progeny observed in the BC_3_/BC_4_ self-populations would be homozygous for the 2S chromosome segment. Alternatively, if the 2S chromosome segment was not preferentially transmitted through the male or the female gametes, then the progeny would segregate for the presence of either one or two 2S chromosome segments. All progeny observed carried two copies of the 2S chromosome segment (Fig. 5), thus demonstrating that the 2S chromosome segment was preferentially transmitted through both the male and female gametes.

**Table 4.** Transmission frequencies of the seven linkage groups of *Ae. speltoides* in the backcross populations (BC_1_ to BC_5_) analysed by SNP genotyping

	BC_1_	BC_2_	BC_3_	BC_4_	BC_5_	Total
Number of plants genotyped	22	252	123	117	22	536
Linkage group 1 (%)	18 (82)	124 (49)	36 (29)	13 (11)	3 (14)	194 (36)
Linkage group 2 (%)	22 (100)	252 (100)	123 (100)	117 (100)	22 (100)	536 (100)
Linkage group 3 (%)	18 (81)	135 (54)	27 (22)	20 (17)	0 (0)	200 (37)
Linkage group 4 (%)	15 (68)	89 (35)	23 (19)	7 (6)	0 (0)	134 (25)
Linkage group 5 (%)	19 (86)	162 (64)	54 (44)	32 (27)	6 (27)	273 (51)
Linkage group 6 (%)	17 (77)	115 (46)	31 (25)	25 (21)	0 (0)	188 (35)
Linkage group 7 (%)	13 (59)	80 (32)	15 (12)	9 (8)	0 (0)	117 (22)

The frequency of transmission of chromosome segments from other linkage groups in each of the backcross progenies was much lower than that observed for linkage group 2. Of the remaining linkage groups, segments from linkage group 5 of *Ae. speltoides* had the highest frequency of transmission in all the backcross progenies observed (Table 4)

## DISCUSSION

*Aegilops speltoides* is a potentially important source of genetic variation for a range of agronomically important traits (Jia *et al.*, 1996; Mago *et al.*, 2009; Klindworth *et al.*, 2012; Deek and Distelfeld, 2014). In the past, it has been difficult to detect and characterize wheat/wild relative introgressions due to the absence of high-throughput, genome-wide marker technologies. In this paper we have utilized an Affymetrix SNP array (King *et al.*, 2017) complemented by a specific crossing strategy and the use of an automated, high-throughput microscope system for GISH image capture.

In this work we generated *F*_1_ interspecific hybrids between wheat and *Aegilops speltoides* and backcrossed these to wheat (Fig. 1). Hybrids were haploid for the A, B and D genomes of wheat and the S genome of *Ae. speltoides*. Thus, in the absence of homologous chromosomes the only recombination occurring at meiosis was between homoeologous chromosomes. This would normally be prevented by the presence of *Ph1*. However, the presence of the pairing promoters *Su1-Ph1* and *Su2-Ph1* located on chromosomes 3S and 7S of *Ae. speltoides* (Chen and Dvorak, 1984; Dvorak *et al.*, 2006) enabled homoeologous recombination to occur.

Since the interspecific *F*_1_ hybrids generated were haploid for the A, B, D and S genomes, their fertility was predicted to be low and this was found to be the case (Table 1). However, their fertility of 29 % was higher than that previously observed in those between wheat and *Amblyopyrum muticum*, which was 16.2 % (King *et al.*, 2017). The low fertility of the *F*_1_ hybrids resulted in the generation of only 22 BC_1_ seeds that grew to maturity and set seed. Hence, if recombination did not occur in later generations, i.e. in the gametes of the BC_1_, BC_2_, BC_3_ and BC_4_ generations, then the total number of introgressions that could be generated was limited to the 22 female *F*_1_ gametes giving rise to these 22 BC_1_ plants. However, the level of interspecific recombination detected by the genetic mapping was such that it was possible to assemble the seven linkage groups of *Ae. speltoides*. Using the genetic linkage map of *Ae. speltoides*, we estimated that 294 wheat/*Ae. speltoides* introgressions were generated spanning the entire *Ae. speltoides* genome. We were able to characterize these introgressions and track them through the backcross generations (Fig. 3). However, while using the genetic map to characterize the introgressions, it was important to treat the cM distances with caution, as the maps were not produced using proper mapping families. Tracking the introgressions through the different backcross generations via the SNP data showed that the majority of introgressions generated had occurred due to recombination in the *F*_1_ gametes and were therefore present in the BC_1_ plants.

Validation of introgressions identified by SNP analysis was carried out by GISH analysis. In most cases the number of wheat/*Ae. speltoides* introgressions detected by the SNP analysis corresponded to the number of introgressions detected by the GISH analysis (Table 3 and Fig. 3). The one exception (BC_5_-4A) can be explained in two possible ways. The first possibility is that linkage group 2S of *Ae. speltoides* has recombined with a wheat chromosome. Although the telomere is on a separate chromosome, the SNP markers would have detected the two introgressions as a complete chromosome. The second possibility is that the genetic linkage map of *Ae. speltoides* is not complete and therefore the smaller introgression has not been detected at all and the large segment has appeared complete in the SNP analysis.

**Fig. 6.**
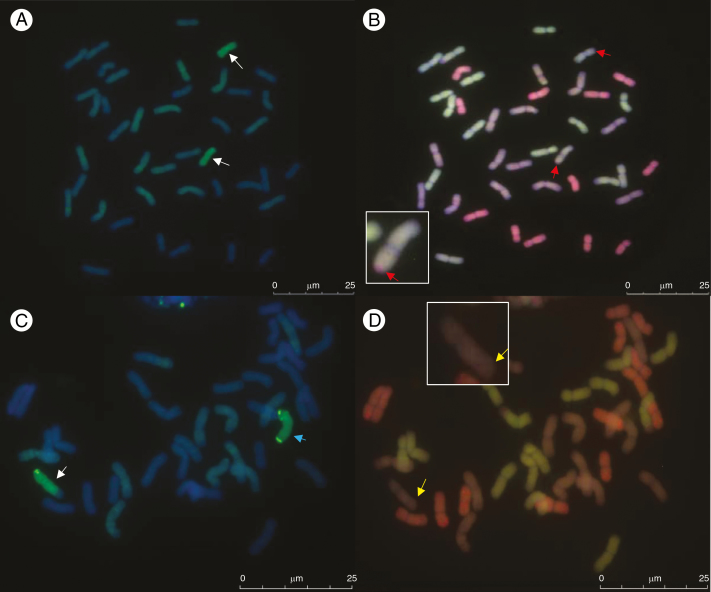
Genomic *in situ* hybridization showing recombination between *Ae. speltoides* and the B and D genomes of wheat. (A) GISH analysis of a 41-chromosome BC_3_*F*_1_ individual. Metaphase spread with labelled genomic *Ae. speltoides* DNA as probe showing *Ae. speltoides* (green) introgressions (white arrows). (B) Same metaphase spread as (A) with three-colour GISH showing *Ae. speltoides* recombined with the D genome (red arrows). Enlarged inset shows one of the recombined chromosomes. This individual carries 14 A chromosomes (green), 13 B chromosomes (purple), 12 D chromosomes (red) and two wheat/*Ae. speltoides* 2S recombinant chromosomes (the segment of the D genome is red and marked with the red arrow). (C) GISH analysis of a 42-chromosome BC_5_ individual. Metaphase spread with labelled genomic *Ae. speltoides* (green) DNA as probe showing one complete *Ae. speltoides* chromosome (blue arrow) from linkage group 2S and one wheat/*Ae. speltoides* recombined chromosome (white arrow) from *Ae. speltoides* linkage group 5S. (D) Same metaphase spread as (C) with three-colour GISH showing *Ae. speltoides* recombined with the B genome (yellow arrow). Enlarged insert shows the recombined chromosome. This individual carries 14 A chromosomes (green), 12 B chromosomes (purple), 14 D chromosomes (red), one complete *Ae. speltoides* chromosome (purple) and one wheat/*Ae. speltoides* recombinant chromosome (purple).

Multicolour GISH analysis was carried out to verify which of the wheat genomes were involved in recombination with *Ae. speltoides* (Fig. 6). The majority of recombination events were shown to have occurred between *Ae. speltoides* and the B genome of wheat (91 %). Recombination had also occurred between *Ae. speltoides* and the D genome (18 %) but we found no evidence of recombination between *Ae. speltoides* and the wheat A genome. As the B genome progenitor of wheat is thought to be *Ae. speltoides* or a close relative (Sarkar and Stebbins, 1956; Riley *et al.*, 1958; Dvorak and Zhang, 1990; Maestra and Naranjo, 1998), the higher level of recombination between *Ae. speltoides* and the B genome was expected. The low level of recombination observed with the D genome of wheat would also suggest that *Ae. speltoides* is more closely related to the D genome than to the A genome. This situation closely mirrors that observed between wheat and *Amblyopyrum muticum*, where most of the recombination occurred between the chromosomes of the wild relative and the B and D genomes of wheat (King *et al.*, 2017). In addition, although the genetic location of the *Ph1* pairing suppressors located on *Am. muticum* are not currently known, the fact that both *Ae. speltoides* and *Am. muticum* carry suppressors indicates that the two species may have had a common ancestry.

We are currently unable to use the SNP markers to identify which of the chromosomes of wheat have recombined with the *Ae. speltoides* chromosomes. We are currently developing wheat chromosome-specific Kompetitive Allele Specific PCR (KASP) markers from the SNP markers on the 35K array. These markers will allow the analysis of large numbers of individuals, firstly to tag individual introgressions and track them through the generations (both via backcrossing and selfing) and secondly to identify which wheat chromosome(s) is (are) involved in each introgression once homozygous introgressions have been generated. Although considerably more labour-intensive and technically demanding, the latter could also be achieved using fluorescence *in situ* hybridization (FISH) with repetitive DNA sequences combined with GISH. This has been successfully demonstrated to identify the recipient wheat chromosomes with introgressions from *Thinopyrum bessarabicum* (Patokar *et al.*, 2016), *Th. intermedium* and *Secale cereale* (Ali *et al.*, 2016) and *Aegilops markgrafii*, *Aegilops triuncialis* and *Aegilops cylindrica* (Molnár *et al.*, 2015).

In the *Ae. speltoides* accession used in this study, chromosome 2S was found to be preferentially transmitted at a frequency of 100 % through both the male and female gametes. This demonstrates that the gametocidal genes on chromosome 2S are extremely potent and resemble those in species such as *Aegilops sharonensis* (King et al., 1991a, b; King and Laurie, 1993). The transmission of the other six linkage groups of *Ae. speltoides* was also observed in the female gametes, and although linkage group 5 showed a potentially elevated rate of transmission, there is no evidence at this time to suggest that it carries a gene for preferential transmission.

It is important to note that the work undertaken was based on a single accession of *Ae. speltoides* and thus it is not possible to determine whether all other accessions carry gametocidal genes on chromosome 2S or other linkage groups. However, if they do, then it will be necessary to remove them if introgression lines without them are to be developed. In order to develop lines that lack the 2S gametocidal genes, but still carry introgressions from other linkage groups, we are currently undertaking research to mirror the work of Friebe *et al.* (2003), who successfully deleted the genes responsible for preferential transmission in *Ae. sharonensis*.

## CONCLUSION

Researchers have been working to transfer genetic variation from its wild relatives into wheat for many years. However, although there have been some notable successes, we have barely scratched the surface of the genetic variation available for exploitation. This has been as a direct result of: (1) the difficulty of inducing wheat/wild relative introgressions and (2) an inability to detect the introgressions. The data obtained here clearly demonstrate that *Ae. speltoides* chromosomes recombine with the chromosomes of wheat at a very high frequency, giving rise to large numbers of introgressions in derivatives of the *F*_1_ hybrid. Considering the importance of the genetic variation identified in *Ae. speltoides* for a range of agronomic traits (Jia *et al.*, 1996; Mago *et al.*, 2009; Klindworth *et al.*, 2012; Deek and Distelfeld, 2014), wheat/*Ae. speltoides* introgressions have the potential to play a critical role in the development of superior wheat varieties in the future.
